# Incorporating a mobile application to support communication about HPV testing among women and professionals: barriers and facilitators from the perspective of health professionals in a middle- and low-income setting in Argentina

**DOI:** 10.3332/ecancer.2024.1778

**Published:** 2024-09-25

**Authors:** Lucila Szwarc, Paula Frejdkes, Victoria Sánchez Antelo, Melisa Paolino, Silvina Arrossi

**Affiliations:** 1 Center for the Study of State and Society, Buenos Aires C1173, Argentina; 2 CONICET National Scientific and Technical Research Council, Buenos Aires C1414, Argentina

**Keywords:** mobile health, HPV testing, cervical cancer, implementation science, health professionals, low- and middle-income countries

## Abstract

**Introduction:**

The delivery of positive Human papillomavirus (HPV) test results can have a psychosocial impact and act as a barrier for women to continue the cervical cancer (CC) prevention process. A previous formative research based on a woman’s perspective indicated that a mobile app could be an acceptable and appropriate tool to help women obtain information and reduce fears related to a positive result. Based on these findings, we developed an app-based intervention that would function as a support for professionals who offer the HPV test and communicate their results. We report data on the perceptions of healthcare providers regarding the barriers and facilitators to the incorporation, in a low and middle-income context.

**Methods:**

Qualitative study based on individual semi-structured interviews with health professionals. All the professionals (*n* =13) took HPV and Pap test samples and provided information on HPV testing, in the public health system of Ituzaingó, Greater Buenos Aires, Argentina. The themes explored were selected and analysed using domains and constructs of Consolidated Framework for Implementation Research (CFIR).

**Results:**

Practitioners had a positive assessment of the intervention through most included constructs: adaptability, compatibility, complexity, relative advantage, belief in the validity and robustness of the intervention, innovation source and knowledge and beliefs about the intervention. However, some potential barriers were also identified including: adaptability, tensions for change, relative priority and leadership engagement. Practitioners conditioned the intervention’s success to specific adjustments of the app (weight and interface usability), legitimmated institutions’ support, and clear and sustained health authorities’ commitment and directions.

**Conclusion:**

Health professionals had a positive assessment of implementing an app to support the HPV test communication and information provision process, although they conditioned its effectiveness to specific adjustments. The results allow us to identify and develop recommendations for the app to be implemented effectively and sustained over time. The findings of this study have important implications not only for Argentina, but also for other low and middle-income countries, given that the implementation could be adapted, with the aim of improving communication between patients and health institutions in the CC prevention process.

## Introduction

Cervical cancer (CC) is one of the leading causes of death in women in most low- and middle-income countries. In Argentina, 4,500 new cases are diagnosed each year and 2,300 women die from this disease [1]. Although CC is preventable with existing knowledge and technologies, the high mortality is due to persistent problems in the cancer control continuum, which condition adherence to it [2–4]. This includes procedures and contacts with the health system over a certain period of time and involves up to four stages: screening Human papillomavirus (HPV test, Pap test), diagnosis (colposcopy, biopsy), treatment and subsequent follow-up [5].

The failures in the CC prevention process respond to socio-structural, subjective-symbolic and institutional factors, including failures in communication between patients and health services [6–9]. The delivery of positive HPV test results (HPV+) can have a psychosocial impact on patients, arousing feelings such as anxiety, fear and shame, given that it is related to sensitive issues linked to sexuality, illness and death [10–12]. This can act as a barrier for women to continue with the process of care in health services [10]. WHO recommends individual counseling, starting with the HPV test and beyond, in which the patient can receive information in clear and simple language, and have time to reflect and express her doubts and fears [10]. However, face-to-face counseling involves a lot of time and resources, which are sometimes not available, particularly in low- and middle-income countries [11, 13, 14], such as trained providers, and sufficient time and privacy during consultations [15, 16]. As a result, women receive insufficient and/or confusing information about testing and its benefits, and how to follow up [8, 13].

In this context, there is a need to develop innovative strategies, applicable on a large scale, to improve the provision of information, counseling and support to women or other sex-gender identities who undergo HPV testing^1^, allowing for more efficient use of resources and enhancing autonomy with patient-centered information.

MHealt (mobile health) strategies can be an important resource to improve information provision and communication between users and healthcare institutions, enhanced by the changes in this field that occurred during the COVID-19 pandemic. The use of mobile applications (apps) to communicate with and support patients has been shown to improve health goals for different conditions, including mental health conditions [20–22]. Apps can be used even after the consultation is over and require fewer staff [20, 23]. In oncology care, they offer the ability to provide accessible information and education at minimal cost throughout the care continuum [24, 25].

In CC prevention, research developed in Argentina demonstrated the effectiveness of MHealth strategies to increase the coverage of Pap triage, a necessary step after HPV+ to identify if the woman needs diagnosis and follow-up [26]. Along these lines, our research team initiated a project aimed at designing an app to improve communication between professionals and patients, and thus reduce the psycho-social impact of HPV testing [27, 28]. In the framework of this research, women users indicated that they would use this technology if it were recommended by a professional and that it would be a good complement to the HPV consultation, allowing them to obtain information and reduce fears related to a positive result [28]. Based on these findings, we proposed an intervention in which health professionals recommend the app to their patients, being an active part of the implementation of the innovation. The aim is for the app to function as a tool to support the work of professionals who offer the HPV test and communicate its results to women.

According to a systematic review [29], the implementation of apps during the care process is perceived by health professionals as a facilitator of communication with patients and between colleagues, coordination and quality of care, as well as the recording of patient information. The research presented in this paper examines the perception of health professionals on the incorporation of a mobile application as a strategy to strengthen communication between patients and health services in the process of CC prevention. In particular, their perceptions about possible barriers and facilitators to the implementation of the mobile application are reviewed.

From the implementation sciences approach, it is essential to know both the point of view of the actors involved (workers and patients) and the possible conditioning factors to the intervention, in order to develop effective strategies for the implementation of the innovation [30]. Recognising the perspective of health professionals allows the consideration of a central actor for the innovation to be implemented, sustained over time and effective as a tool for articulation between women and professionals [28] This will also allow us to identify barriers and facilitators to implementation, in order to develop strategies in the aforementioned directions.

## Material and methods

### Framework

The project is framed within the implementation sciences and the theory of diffusion of innovations [30, 31], which provides conceptual tools for understanding the adoption, dissemination, diffusion and implementation of innovations in the field of health. We rely on the Consolidated Framework for Implementation Research (CFIR), which allows us to take into account the multilevel factors that can condition the success of an implementation, based on a wide range of constructs [30].

The CFIR constructs have been associated with the effective implementation of innovations and are organised into five domains: 1. The aspects related to the characteristics of the innovation and that will shape its implementation; 2. The external environment, which comprises the social, political and economic situation of the organisation in which the innovation will be implemented; 3. The organisational or internal environment, which includes the political, cultural and structural atmosphere through which the innovation will be processed; 4. The characteristics of the people involved in the implementation of the innovation (facilitators such as authorities, adopters and recipients); and 5. Table 1 presents the constructs that were selected and used in this research, within each domain.

### Scope of research

The work was carried out in the province of Buenos Aires, in the municipality of Ituzaingó, where the formative research had previously been developed with women. This municipality established in 2015 the HPV test as primary screening for women aged 30 years or older, users of the public health system, not covered by social security. The locality has six primary health care centers, linked to the second level, which offer free care, including detection, diagnosis and treatment if necessary.

Thirteen professionals from the public health system, dedicated to the prevention of CC in the municipality, whose tasks include the provision of information on HPV testing, were interviewed.

### Data collection

Individual semi-structured interviews were conducted with health professionals through the videoconferencing platform (Zoom Inc). Currently, the use of Information and Communication Technologies is accepted and valid for the collection of information, because it allows greater flexibility for a synchronous encounter, without losing certain qualities of a face-to-face meeting between informant and researcher [32]. The interview guide was organised based on specific CFIR constructs (Table 1).

To provide more clarity on the proposed implementation, during the interview, it was explained that the app would be a tool to support the work of professionals who offer HPV testing and communicate their results. A presentation with the app’s key screens was also shown.

With prior authorisation, the interviews were recorded, with an average duration of 40 minutes. The interviews were transcribed and individual codes were assigned to protect the anonymity of the participants.

### Data analysis

Data coding and analysis were performed according to the selected domains, constructs and dimensions of the CFIR (Table 1). Thematic analysis was used to identify emergents, classified as barriers and facilitators. The analysis was performed by two researchers, and then discussed together with the research team. To ensure the internal consistency of the coding, the constant comparison strategy was used and disagreements were resolved by reviewing the original data [33].

### Ethical aspects

Ethical standards were in accordance with the 1975 Declaration of Helsinki on human experimentation. The project and the informed consent were approved by the Ethics Committee ‘Diagnóstico por Imagen Morón’ with registration number N°060/2016. Express informed consent was requested from all interviewees to participate in the study and to audio record the interviews, where full respect for anonymity and confidentiality was indicated.

## Results

### Sample characteristics

Thirteen health professionals were interviewed, 4 men and 9 women, of whom 9 were gynecology professionals, 1 general practitioner, 2 obstetrics graduates and 1 proctology professional, in charge of HPV navigation. All the professionals work in primary health care, where they take HPV and Pap test samples and provide information on HPV testing.

Table 2 summarizes the results based on the CFIR constructs as perceived as barriers or facilitators of app implementation, with verbatims for each construct and subtheme. They are presented according to the order of dimensions and constructs proposed by the CFIR.

### Facilitators for the incorporation of a mobile application in CC prevention

From the analysis of the interviews, we found that, according to the professionals, seven CFIR constructs could function as facilitators when implementing the app (see a summary of the results and interview excerpts for each construct in Table 2). It is perceived that the app would be compatible with the current practice, given that technological tools are being used for communication with patients (such as institutional mail or WhatsApp) and the cell phone is being used in the framework of the consultation, either to access results in computerised records or to see those brought by women. Likewise, the people interviewed consider the app ***adaptable*** to the current way of providing information, in that it would not replace the consultation, but would complement it, so that women could, for example, clear up doubts that arise later or reorient their questions based on information from the app. Along these lines, the app is also perceived as easy to incorporate (low complexity), because it would not take much time to mention it or suggest downloading it during the meeting.

The compatibility is linked to and reinforced by the relative advantage that the app provides, in relation to other possible ways of providing information. Currently, according to professionals, women do not have information about HPV, and this leads them to search the internet where false and confusing information circulates. The app, then, would be a good tool to offer them where to look for reliable online information, instead of discouraging them from doing so, as professionals do today. Two arguments that enhance the positive perception of the relative advantage stand out: the fact that the information is available, ‘at hand’, i.e., that it is compatible with the widespread use of cell phones and, in turn, that it is supported and validated information. The latter represents an important relative advantage over information circulating on the Internet, which could negatively affect women’s perception of CC. The people interviewed also consider the advantage of being able to offer the app to those professionals who do not provide information on HPV, due to lack of training or because they only indicate the next steps in the preventive process.

Respondents believe that there is ***evidence of the validity and robustness*** of the effectiveness of implementing an app. Although they do not mention other experiences of using mobile applications to provide preventive information in the health system, the widespread use of the CUIDAR app, officially implemented in Argentina during the COVID-19 pandemic, is mentioned as a positive precedent. This app was widely used to register vaccinations, and to request and provide application appointments and circulation permits.

Regarding the ***origin of the intervention***, the people interviewed support the app because they know the team conducting the research on its development and implementation (CEDES, Centro de Estudios de Estado y Sociedad) and mention their prestige and experience, together with those of the public institutions that financed the research, such as the National Cancer Institute of Argentina. Thus, as regards the construct that reflects the ***belief in the intervention*,** the people interviewed indicate that they would accept the app in order to recommend it to their patients, and believe that it would be accepted by the other members of the healthcare team, as it is promoted and supported by recognised and legitimized institutions and experts.

### Potential barriers to the incorporation of mobile applications in QC prevention

Four CFIR constructs were associated with possible barriers to implementation. The first barrier refers to ***adaptability****.* Although the people interviewed describe that patients use apps, they also emphasize that, in order to adapt to the local context, the app design should require little memory on the cell phone and provide a user-friendly interface. They also understand that every app has a limitation in terms of content, so it would not be able to answer all possible questions.

The people interviewed have a very positive perception of how they provide information on HPV and CC prevention: they describe multiple communication strategies to ensure that women understand, that they have no doubts and that they take the necessary time to do so. Some of them also consider that, despite certain limitations in terms of available resources, the professionals are sufficiently trained to inform patients. In this sense, they do not perceive the need to implement changes in the way information is provided (***Tensions for change***). However, the interviewees also reported that women do not know what the HPV test is and that it is essential to explain and reinforce the information repeatedly. In this sense, they describe failures in communication, which can be recognised as the interviewees’ own, or that of other professionals who focus only on giving follow-up instructions, as can be seen below:

*‘It seems to me that the fault lies in reading the booklet and saying ‘well, you have HPV, the Pap was positive, you will have to have a cone’ (...) it seems to me that the best thing you can do is to make her understand you and where we fail, we fail.’ (E5)*

Linked to these problems, they also describe an environment that makes communication difficult, due to work overload or lack of time and privacy for consultation. The following testimony is exemplary:

*‘In the office we can have an overload of patients. In other words, we can’t stop much in terms of information (...) we do everything quickly (...) it is a very small office, the door has no lock, we have to lock it with a stool. Inside the office there is also a bathroom shared with colleagues. So when I’m in the office, a colleague might knock on the door to let me in...’ (E4).*

They also consider that an app would be an important intervention but not necessarily a priority (***relative priority***). However, they agree that CC is a serious problem that should not exist, and that part of the problem is related to patients’ lack of information and emotions such as embarrassment and fear of screening. In this sense, they consider that an intervention such as this could contribute to disseminating information and improving adherence.

Finally, the interviewees argue that the way the app is implemented would be substantial for the intervention to be successful. In this sense, they believe that all people working in the health center should be involved. They also highlight the importance of the role and commitment of the ward manager, along with the implementation of clear, even mandatory, guidelines (***Commitment of the authorities***). In general, they consider these commitments and guidelines to be a prerequisite for successful implementation.

## Discussion

It has been documented that, during the CC prevention process, there are problems in communication between professionals and patients that could affect the continuity of the care process [8, 7]. To the best of our knowledge, there is no research on the development of an app aimed at improving the process of information and communication with patients, with the exception of the study conducted by our team focused on women [28]. This is the first research focused on the perspective of health professionals and key actors to ensure the effectiveness of the implementation. We followed the guidelines of the CFIR, an appropriate conceptual framework for evaluating the implementation and sustainability of health interventions [30]. Evidence suggests that incorporating theoretical approaches into implementation research in the field of public health and clinical practice can improve the diffusion and use of digital technologies [34, 35].

The results show that the professionals evaluate the app positively, which is expressed in the CFIR constructs: adaptability, compatibility, complexity and relative advantage of the intervention in relation to the current context, belief in the validity and soundness of the intervention and its origin, and knowledge and beliefs about the intervention. In turn, they identify potential barriers corresponding to the constructs: adaptability, tensions for change, relative priority and authorities’ commitment to implementation.

The barriers perceived by professionals refer, in the first place, to issues of adaptability of the innovation, such as the weight of the app and ease of use. This coincides with other studies that find accessibility barriers to mHealth interventions aimed at patients (during the intervention or pre-implementation, from the professional point of view), such as clarity in its use, or access to a cell phone and Internet signal by users [36–38]. In our research, another barrier pointed out in relation to adaptability is the fact that the app could not answer all possible questions from women, but only a finite list of doubts. Based on these and other observations, recommendations for the potential development of the app are presented in Table 3. Several research studies focused on users, health workers and/or decision-makers allowed the development of recommendations for cell phone technologies, adapted to local needs, barriers and facilitators [36, 39, 40].

Beyond the aspects related to the app design, the barriers mentioned refer mainly to the internal context. Two main barriers were identified: on the one hand, the interviews do not consider implementation as a high priority, in relation to other issues and, on the other hand, they do not identify the current situation as intolerable or in need of change. However, these two perceptions are nuanced. Although professionals do not consider the app as a priority, they do consider it important, and see CC as a serious problem that needs to be addressed urgently. In this sense, they see the app as an instrument that could effectively contribute to the prevention of CC. At the same time, although they describe their task in communicating about HPV positively, they also find failures in communication, linked to their own role, and that of their colleagues, and to environmental barriers, such as lack of time, work overload or lack of privacy during the consultation. These communication problems, reported by several studies carried out in Latin America [11, 13], would be a sufficient reason to make modifications or incorporate elements that contribute to improving the current situation.

In addition to the above-mentioned nuance regarding relative priority and tensions for change, it is also important to note that the app is perceived as advantageous in relation to the way in which information is currently provided. According to a systematic review of health innovations [31], recognition by stakeholders of the relative advantages of an intervention is a prerequisite for its adoption. Along the same lines, research conducted in Canada found that one of the main factors that facilitated the implementation of a mobile application for monitoring cardiac patients was that physicians perceived it as advantageous with respect to other telemonitoring systems [41].

The relative advantage is related to the professional perception that the app could complement, improve and reinforce the current situation in terms of doctor-patient communication and information received by women in general. In a study conducted in Kenya on the implementation of a mHealth strategy based on text messages to improve adherence of people living with HIV, it was also found that, according to professionals and users, the intervention could be a tool to improve the relationship between patients and health institutions. As in our research, several studies have found that mobile health applications can increase patient empowerment, generating greater connection with the medical team between consultations and increasing access to medical information [42, 43]. These findings are fundamental in middle- and low-income settings, given that mHealth interventions could improve the quality of services and health *outcomes in* a cost-effective manner, in the short or medium term, in settings with strong structural inequities in health, which are much more complex and costly to address [42, 43].

The last barrier mentioned refers to the commitment of the authorities as a prerequisite for successful implementation. Again, this is a result that allows recommendations and adaptations to be made to the way the app is implemented, but it is a recommendation that is particularly dependent on the local context. Research conducted in Argentina on the scale-up of a mHealth strategy to improve adherence to *screening* found that, although collaborative work had been done to plan scale-up activities, each change of health authority had slowed the actual incorporation of the strategy as a routine programmatic activity [44]. In our case, both the research focused on women and the present one was carried out with the support of municipal and provincial health authorities that would have stability in the short term. However, it is essential, when implementing the app, to work towards retaining the support of the different authorities in order to generate clear guidelines for implementation, as well as to generate evidence on possible strategies for effective implementation in contexts of high turnover of health authorities (Table 3).

In general terms, it can be said that the barriers identified are minor and manageable. On the one hand, they are mainly counteracted by the professional perception (priority, relative advantage). On the other hand, it is possible to formulate specific, uncomplicated recommendations and adaptations for the development of the implementation. Evidence shows that when stakeholders are included, from recent stages of intervention development and during the process itself, the intervention is seen as more feasible, effective and potentially successful [25, 36]. It should be recalled that the results presented here seek to complement those of a previous survey, focused on patients’ perceptions. There, women indicated that they would use the app to obtain information on HPV and reduce fears linked to a positive result, if it were recommended by a professional or a health authority, in addition to indicating app format preferences that also allowed establishing guidelines for the intervention [28].

With respect to the facilitators, the professionals find that, in addition to being advantageous, the app is seen as adaptable to local needs and not very complex to implement, domains related to the characteristics of the intervention. They also perceive it to be compatible with the local internal context, in that it could be implemented in the practice, without the need for modifications to current practice. This is consistent with research conducted in Germany, in which about 85% (*n* = 108) of clinicians surveyed felt that an app for oncology patients could complement traditional care and treatments [28]. More recent research evaluating successfully adopted mHealth implementations also found facilitators in adaptability, compatibility and other constructs related to intervention characteristics, as well as compatibility with current practice (internal context) [36, 41].

The people interviewed indicated that they would accept the app in order to recommend it to their patients and believe that it would be accepted by the other members of the health team (knowledge and beliefs about the intervention), as it is promoted and supported by recognised and legitimized institutions and experts (origin of the intervention). Among them, they mention the team developing the research on the implementation of the app (CEDES, Centro de Estudios de Estado y Sociedad) highlighting their prestige and experience, together with those of the public institutions that financed the research, such as the National Cancer Institute of Argentina**.** This is an important finding given that the legitimacy of the origin of an intervention is also strongly related to the success of its implementation [45]. The research focused on professional perspectives on health app implementations (in general medicine and oncology) and found that trust in the source that promotes or endorses the app is the main facilitator or requirement for professionals to implement the app [46, 47]. The professionals interviewed also consider that there is valid and solid evidence to support the effectiveness of implementing the app. They mention as an important precedent an app used during the COVID-19 pandemic, recommended by the National Ministry of Health of Argentina. The mention of an effective and valid antecedent according to the professional perspective is also a key data, since it could work as an important facilitator.

## Limitations

One limitation of this study is that it evaluates the professional perspective in a specific context, which limits the generalisability of the results. In addition, this is an evaluation conducted prior to the development of the app under study. In this sense, further studies should be conducted during the development and implementation of the app. Future research will also be needed to adjust the intervention to other health systems, contexts and local needs.

## Conclusion

The results of our research indicate that health professionals who offer the HPV test and communicate their results to women see the implementation of an app aimed at improving the process of information and communication with patients as positive, advantageous, viable and legitimate. With great acceptance, they indicate that they and their colleagues would implement it, although they condition the success of the intervention to certain aspects. The results allow the identification of barriers and facilitators and the elaboration of specific recommendations so that the app can be implemented, sustained over time and be effective as a tool for articulation between women and professionals.

The results obtained allow a better understanding of the factors that favour the implementation of mHealth interventions that seek to complement medical consultation. They have important implications, in particular, for low- and middle-income countries, given that the implementation could be adapted to other contexts, in order to improve communication between users and health institutions and the information of women in the CC prevention process.

## List of abbreviations

CC, Cervical cancer; CEDES, Center for the Study of State and Society; CFIR, Consolidated Framework for Implementation Research; HPV, Human papillomavirus;mHealth, mobile health.

## Conflicts of interest

The authors declare that there are no conflicts of interest.

## Figures and Tables

**Table 1.** Dimensions, domains, constructs and definitions of CFIR used.

Dimension	Construct	Definition
Possible facilitators		
Domain I: Innovation	Belief in the validity and soundness of the intervention.	Belief that there is valid and solid evidence to support the effectiveness of implementing the app.
	Origin of the intervention	Legitimacy of the person who promotes the implementation of the app, as an external figure to the institutions that implement it.
	Relative advantage of the intervention	Perception of implementing the app in relation to other current or possible interventions.
	Adaptability of the intervention	Perception of the app implementation as adaptable or perfectible in relation to local needs.
	Complexity of the intervention	Perception of app implementation as complicated, which may be reflected in its scope and/or the number and type of steps required.
Domain III: Internal context	Compatibility of the intervention	Perception of implementing app as compatible with current practice.
Domain IV: Characteristics of individuals	Knowledge and beliefs about the intervention	Individuals' attitudes towards the app and the value they place on it, whether they would recommend it to their patients, and whether they believe it would be accepted by healthcare teams.
Possible barriers		
Domain I: Innovation	Adaptability of the intervention	Perception of the app implementation as adaptable or perfectible in relation to local needs.
Domain III: Internal context	Tensions for change	Perception of the current situation as intolerable or in need of change.
	Relative priority	Shared perception of the importance of implementing the app.
	Commitment of the authorities	Who should be involved and committed to the implementation of the app: the center, the network of centers, the municipality and others.

Prepared by the authors based on the adaptation of Damschroder [30]

**Table 2.** Dimensions, CFIR constructs and interview excerpts, analysed as barriers or facilitators.

Dimension	Construct	Subtopics	Verbatims
Possible implementation facilitators			
Domain I: Innovation	Belief about the validity and soundness of the intervention.	Valid background	‘Just as the CuidAr app was created when we were in the middle of the pandemic, when you had to get a certificate of circulation, or check the symptoms to get on a means of transport (...)’ (E6).
	Origin of the intervention	Legitimized and supported origin	‘(...) but in this case, which comes from you (the research team), I think so. (...) it will be easy for us because we already know the support that this app has because we have been working with you.’ (E8)
	Relative advantage	Accessibility of reliable information	‘(...) that any woman has access to information, backed up, not that she told me, I don't know, the one on Instagram (...), because the lady can easily at any place, at any time, or even on a bus ride, 'oh I'm going to see what this is about’ (E7).
		In relation to internet searches	‘(...) the only information they can get about HPV, real information, because in reality you google and you will google from that if you have HPV you have cancer to a thousand other things, but real information is what we can provide in the little time, short time we have in a small room.’ (E9) ‘(...) many times they come scared because they looked and saw everywhere and (only) when they come we tell them that they have nothing’ (E3).
		In relation to discouraging Internet searches	‘Patients google a lot, so, we are always saying 'don't google'. Well, you could say, 'look at this, it's information that doctors wrote, don't google, go this way'’. (E8)
	Adaptability	Adaptable to the consultation in which information is provided (complements for guidance)	*‘If there are things that remain unresolved, they can go on probing from there, or reinforce what they were told in the consultation. The information is reinforced and at the most, if they come for a next check-up, they will already have a clearer idea of the subject and be able to direct their specific question, because sometimes they may have many doubts, but they cannot formulate the exact question.’ (E7) ‘(...) if you see that a patient has many doubts or something, you tell her ‘look, you have the application, you have the facility to get to the information, and if you have any more doubts, we will solve them at the next visit’ (E7).*
		Adaptable to situations where professionals do not provide information	*‘So, the one who does not like to explain, (...) is going to tell the patient 'Go and look for the app'.’ (E10)*
	Complexity	Low complexity (easy to incorporate)	*P: Yes (it can be proposed in the consultation). Because it would not take much to explain all this’ (E4).*
Domain III: Internal context	Compatibility	Using the Internet for communication	‘We have a mail that in pandemic arose, (...) I thought it was not going to be very successful and the truth is that we all manage with mail.’ (E8)
		With cell phone use in consultation (professional)	‘Here we use the SITAM (computerised patient registry) (...) because many girls want to repeat it (the pap) and I always have to corroborate that I really have the time and the correct result to repeat it.’ (E7)
		With cell phone use in consultation (patients)	‘(...) everyone sends, (...) the lab results by e-mail, for example, and the pregnant woman sometimes shows it to me from her own cell phone, so, it doesn't seem crazy to me to create something that they can manage from their own cell phone, (...) I think it could work.’ (E6)
Domain IV: Characteristics of individuals	Knowledge and beliefs about the intervention	Acceptance	‘it would be very interesting to incorporate (the app) in the health system, and especially in the part where one works, in the peripheral health centers or (...) to be part of what would be counseling.’ (E4) ‘I would recommend it to my patients] as long as it has the endorsement (...) that there is someone behind it who knows what is being reported, that it is done responsibly (...) why wouldn't I recommend it?’ (E6).
Possible barriers that could condition implementation			
Domain I: Innovation	Adaptability	Design (weight and ease of use as conditioning factors)	‘The app is the medium, now, in order to reach that medium, other issues have to be solved, that the cell phone can download it (...) that it is not so heavy and that it is not so difficult’ (E9).
		Not adaptable to certain questions	‘(...) there will always be questions that the application itself cannot solve for the patient.’ (E7)
Domain III: Internal context	Tensions for change	The following are not perceived	*‘I don't think there is an obstacle in the system; when you have to give information, you give it if you want to. The obstacle is, I see it more in the patient's arrival at the center (...) I think there are not many excuses for not providing information if the patient is already at the center. If you have (resources) it is much better, and much nicer, but if you have nothing, you have the knowledge and you provide it in some way.’* (E8)
		Need for change: lack of information from women	‘Before (taking) I explain to them what we are going to do. Once I have taken (...) I go back to reinforce (...) And then, when she comes back with the result (...). Now, the one I didn't take, I take the trouble to tell her what it is about when she comes back with the result (...), I give her a little talk, because none of them knows what the HPV test is’ (E10).
		Need for change: own failures and those of the environment hinder communication.	*‘(Factors that may hinder providing information on HPV in the consultation itself are) fundamentally time (...) and also the health professional we are, exhausted, and then, that generates that the care may not be the excellence it requires, it is so much information in a consultation.’* (E1)
	Relative priority	Not urgent but important	‘I don't know if it would be a priority, but it is another tool’ (E10). ‘I always say that today no woman should die from CC. (...) because it is totally diagnosable and treatable. Why do they die? Because ten years passed and they did not see a doctor (because of) lack of information, fear, shame.’ (E9)
	Commitment of the authorities	Involve all stakeholders in the system	‘(...) the whole health system has to be involved, otherwise it is useless (...) Everyone should know that this exists, everyone should be able to propose it.’ (E9)
		Clear and mandatory guidelines	‘They would have to train all the doctors if that app is going to come out and tell them 'it's going to do like this, and you have to say this.' (...) half as a tax. (...) it's the only way we're all going to do it.’ (E10)

Prepared by the authors based on the adaptation of Damschroder [30]

**Table 3.** Recommendations for the implementation of the app based on the results obtained, by domains and constructs of the CFIR.

Domains and construct	Possible barrier or condition for success	Recommendation
Domain I: Innovation		
App adaptability	Difficulties for users in downloading the app due to its weight	- Prioritize low storage weight.
	Difficulty for users to understand the app interface	- Design a simple interface with clear language.
	Finite list of questions	- Dialogue channel with users so they can ask questions that the app does not answer (via message box, chat or discussion wall with supervision). - Dynamic content team that can incorporate recurring questions as new answers. - Possibility in the future of developing artificial intelligence to improve the specificity of the responses.
Domain III: Internal context		
Support from the authorities	Support for all system stakeholders	- Develop strategies to involve all health center staff (different levels), together with local authorities.
	Clear guidelines for implementation by all personnel	- Develop incentives and guidelines for medical staff to implement the app in the practice and recommend it. - Evaluate different incentive strategies with local authorities.

Own elaboration based on adaptation of Damschroder [30]

## Footnotes

1 Screening for cervical cancer (HPV or Pap test, depending on availability and indication) should be indicated for all persons with a cervix, whether or not they have had vaginal penetration [17, 18]. This includes women who have sex with women, and non-binary and trans men with a uterus. Given that the literature on CC prevention in people of diversity is sparse and shows that this group goes through particular barriers and experiences [19], we focus on research focused on women. But we do not want to neglect to mention that future research, communication and information provision strategies should also include these population groups.
